# Intermittent Fasting and *Akkermansia muciniphila* Exert Independent and Combined Benefits on Behavioral and Neurobiological Deficits in a VPA-Induced Autism Rat Model

**DOI:** 10.3390/nu18050777

**Published:** 2026-02-27

**Authors:** Emre Adıgüzel, Beyzanur Bağçovan, Nuh Mehmet Bozkurt, Gökhan Ünal, Napoleon Waszkiewicz

**Affiliations:** 1 Department of Nutrition and Dietetics, Faculty of Health Sciences, Karamanoğlu Mehmetbey University, 70100 Karaman, Türkiye; 2 Department of Neuroscience, Gevher Nesibe Genome and Stem Cell Institute, Erciyes University, 38280 Kayseri, Türkiye; 3 Department of Pharmacology, Faculty of Pharmacy, Erciyes University, 38280 Kayseri, Türkiye; 4 Department of Psychiatry, Faculty of Medicine, Medical University of Białystok, 15-272 Białystok, Poland; napoleon.waszkiewicz@umb.edu.pl

**Keywords:** autism, intermittent fasting, probiotics, social interaction, neuroinflammation, apoptosis, autophagy, gut permeability

## Abstract

**Background/Objectives**: Autism is a complex neurodevelopmental condition characterized by social and cognitive impairments, with growing evidence implicating neuroinflammation, disrupted autophagy, apoptosis, GABAergic dysfunction, and gut permeability in its pathophysiology. Thus, this study aimed to evaluate the independent and combined effects of intermittent fasting (IF) and the next-generation probiotic *Akkermansia muciniphila* on behavioral outcomes and molecular markers in prenatal valproic acid (VPA)-induced autism model. **Methods**: Male rat offspring were allocated into five groups (*n* = 8 per group): control, VPA, IF, probiotic, and IF + probiotic. The groups other than the control group were exposed to 500 mg/kg VPA prenatally to establish an autism model. Intermittent fasting (16:8 time-restricted feeding) and *Akkermansia muciniphila* (1 × 10^9^ cfu/day) were applied for 30 days. Behavioral tests (stereotypy, social interaction, memory, and anhedonia) were performed during the last eight days of the treatment period, and the rats were sacrificed the following day for collection of brain tissue and serum samples. Proinflammatory, apoptotic, autophagic, and GABAergic markers were measured in the prefrontal cortex and hippocampus, while zonulin levels were measured in the serum. Data were analyzed using one-way ANOVA followed by Tukey’s post-hoc test. **Results**: Prenatal VPA exposure worsened all behavioral and molecular parameters. All treatments improved stereotypy, social interaction, and memory, whereas anhedonia improved only in the combined treatment group. The treatments also decreased neuroinflammation and apoptosis-related imbalance while enhancing autophagy and GABAergic markers. In terms of apoptosis- and autophagy-related markers, the IF-only and probiotic-only treatments were effective in the prefrontal cortex, while the IF + probiotic treatment showed its effect in both brain regions. Lastly, all treatments were successful in alleviating elevated serum zonulin levels. **Conclusions**: Intermittent fasting and *Akkermansia muciniphila* alleviate VPA-induced behavioral and neurobiological impairments. The combined treatment, in particular, offers stronger and multi-targeted therapeutic potential.

## 1. Introduction

Autism spectrum disorder (ASD) is a neurodevelopmental disorder characterized by behavioral patterns such as persistent deficits in social interaction and communication, repetitive stereotyped behaviors, restricted interests, and sensory sensitivities, according to the *DSM-5* [1]. In addition to the core symptoms, a wide range of behavioral problems may accompany autism, such as anxiety, irritability, decreased cognitive flexibility, impaired learning and memory processes, anhedonia, and sleep disturbances [2,3]. The latest report from the Centers for Disease Control and Prevention (CDC) states that 1 in every 31 children is autistic; this rate has increased over the years, highlighting the critical importance of autism in terms of public health [4].

One of the most notable biological processes in the pathogenesis of autism is neuroinflammation. Evidence is mounting that immune responses triggered by environmental risk factors, particularly during the gestational period, may lead to lasting effects on the fetal brain. It is emphasized that maternal immune activation during the prenatal period may trigger inflammatory and oxidative stress responses in the placenta and fetal brain, which may lead to autism by disrupting neuronal development, synaptic plasticity, and neurodevelopmental programs [5]. Some chemicals may activate brain glial cells—particularly microglia and astrocytes—leading to chronic inflammation, oxidative stress, and synaptic dysfunction and this mechanism may be an important pathway in environmentally induced subtypes of autism [6]. Studies conducted on widely accepted experimental models, such as the valproic acid (VPA)-induced autism model, have also provided findings supporting these biological processes. Rodent models based on prenatal VPA exposure show not only autism-like behavioral phenotypes, but also an increase in inflammatory response in brain tissue, glial activation, and synaptic/structural changes. These findings suggest that neuroinflammation may not only be a “concomitant” phenomenon but also a “causal/facilitating” component in the developmental pathogenesis of autism [7].

In recent years, evidence suggesting that impaired cellular homeostasis, synaptic plasticity, and neuronal quality control processes play a significant role in the etiology of autism has accumulated. Autophagy, in particular, is a critical mechanism for normal neurodevelopment and neuronal sustainability [8], defined as the process of recycling intracellular organelles and proteins via the lysosomal pathway and clearing toxic/worn-out structures. It consists of five stages: as cellular energy decreases, AMPK is activated and mTOR is inhibited, initiating the autophagic flux (initiation). Cytoplasmic debris is enclosed by a double membrane (autophagosome formation), and the resulting autophagosome fuses with a lysosome (fusion). Subsequently, lysosomal enzymes break down the autophagosome contents (degradation). Finally, the resulting amino acids and fatty acids are reused (recycling) [9]. The mTORC1 sensor suppresses the initiation of autophagy by phosphorylating ULK1, while mTORC1 inactivation during energy stress allows for ULK1 activation via AMPK, thereby initiating autophagy. ULK1 is an upstream kinase that phosphorylates and activates the Beclin-1/PI3K-III complex in the early stages of autophagy; this interaction is a fundamental step that initiates the formation of the isolation membrane (phagophore). Beclin-1 is considered the most important protein involved in the initiation of autophagy, and the most critical indicator of autophagosome formation is the conversion of LC3-I to LC3-II [10,11,12]. Recent studies strongly support the notion that autophagy dysfunction is a common and prominent mechanism underlying autism. A meta-analysis encompassing 192 different genetic and environmental autism models reported a consistent reduction in Beclin-1 levels across all autistic subgroups (different types, genders, ages, and brain regions). The decrease in Beclin-1 indicates that autophagic flux is generally suppressed, which may lead to the accumulation of intracellular proteins/organelles or the insufficient clearance of damaged structures. The meta-analysis also indicated a general decrease in LC3-II levels, which supports impaired autophagosome formation and, consequently, impaired autophagy flux [13]. These findings are consistent with neurobiological models suggesting that autophagy is not merely a process associated with stress or starvation but, rather, a continuously active mechanism involved in synaptic pruning, dendritic development, stabilization, and overall neuronal quality control during neurodevelopmental periods [8].

Neuronal apoptosis, which interacts with neuro-autophagy, is also considered an important component of neurodevelopmental pathophysiology. In healthy brain development, apoptosis is a critical process for balancing neuronal numbers, eliminating unnecessary synaptic connections, and forming healthy circuits [14]. Apoptotic mechanisms are disrupted in abnormal neurodevelopmental processes. Apoptosis stands out as one of the underlying cellular processes in neurodevelopmental disturbances observed in individuals with autism and in animal models of autism. Increased pro-apoptotic signals, such as BAX increase and caspase activation, and decreased anti-apoptotic defense mechanisms, such as BCL-2 levels, have been reported in autistic post-mortem brain tissues and various experimental models [15,16]. Imbalances in apoptotic mechanisms can lead to unnecessary or excessive neuronal loss, particularly during the prenatal and early postnatal periods when synaptic formation and pruning are critical. This can disrupt the normal development of brain circuits, contributing to the emergence of social behavior disorders, communication deficits, and repetitive behaviors characteristic of autism [15]. It is thought that apoptosis and factors such as inflammation and oxidative stress can reinforce each other, meaning that cellular stress can cause a multi-layered pathological cycle resulting in both autophagy dysfunction and inflammatory responses as well as cell death [17,18]. This triple interaction (oxidative stress–inflammation–cell death) is increasingly recognized in neurodegenerative and neurodevelopmental conditions [19]. Therefore, although apoptosis-related signaling, as a component of the complex and multifactorial neurobiological processes underlying autism, may potentially contribute to alterations in neuronal survival, synaptic connectivity, and neural circuit development, it is more likely to reflect secondary cellular stress responses rather than a primary driver of pathology [15].

A significant part of neurochemical imbalances in autism is caused by a disruption in the balance between the excitatory glutamatergic system and the inhibitory GABAergic system. Changes in glutamate and GABA levels in different regions of the autistic brain have been frequently reported [20]. The idea that impaired GABAergic inhibition may be associated with autism was proposed following early studies that found decreased GABA receptor expression [21]. Subsequently, significant changes in GABAergic interneuron density in the hippocampus and cerebellum in autism have been reported [22,23]. These observations have contributed to the development of the hypothesis that one of the underlying neurobiological processes of autism may be related to an altered excitation/inhibition (E/I) balance. The primary explanation for the E/I imbalance favoring excitation is the reduced firing of parvalbumin-positive neurons, which constitute the dominant population of GABAergic interneurons [21]. The action potentials in these neurons exhibit characteristics such as minimally adapted high-frequency firing, short membrane time constant, and long hyperpolarization [24]. The *PVALB* gene has been reported to be one of the most prominently downregulated genes in the cerebral cortex of autistic individuals [21]. All of this suggests that functional impairment in parvalbumin-positive neurons underlies the neurobiological basis of autism [25,26,27]. Nevertheless, the molecular mechanisms underlying GABAergic transmission are not fully understood [28].

In recent years, the microbiota–gut–brain axis, which describes the bidirectional communication between the gut microbiota and the brain, has been shown to play an important role in the neurobiological basis of autism. This communication network is a multifaceted structure consisting of the central nervous system (CNS), the autonomic nervous system (ANS), the enteric nervous system (ENS), the hypothalamic–pituitary–adrenal (HPA) axis, and the vagus nerve connecting the gut microbiota and the CNS to the visceral organs [29,30]. Studies on germ-free animals have shown that microbial colonization plays a critical role in the development and maturation of both the CNS and ENS [31,32], and the gut microbiota has been shown to regulate many neurodevelopmental processes in the hippocampus, such as neurogenesis, myelination, synaptic connections, microglia function, and blood–brain barrier permeability [33]. Disruptions in the microbiota–gut–brain axis have been reported to be associated with social interaction problems, anxiety, and neuroinflammatory changes [34,35,36]. For this reason, the effectiveness of probiotic-mediated microbiome modulation on the gut–brain axis has been the subject of numerous studies [37,38,39]. Despite the strong preclinical rationale and promising findings from animal models, the clinical evidence for probiotic supplementation in autism remains inconsistent. Several randomized and controlled trials have reported limited or no significant improvements in core behavioral symptoms following probiotic interventions. While some studies have suggested modest benefits in terms of gastrointestinal complaints or specific secondary outcomes, overall findings indicate that probiotic supplementation alone has not yet demonstrated robust and reproducible clinical efficacy in autism populations. These largely null or heterogeneous clinical outcomes highlight a critical translational gap between experimental findings and human studies, underscoring the need to explore novel strategies, next-generation probiotic candidates, and combination approaches targeting multiple biological pathways simultaneously [40,41]. In light of these translational limitations, attention has increasingly shifted to next-generation probiotics, which may exert stronger and more targeted clinical and biological effects and offer greater potential for the development of targeted therapeutic strategies than traditional strains [42]. *Akkermansia muciniphila*, one of the most notable next-generation probiotics, is an intestinal commensal that targets the mucus layer which, as a critical member of the intestinal microbiota, plays multifaceted roles in energy metabolism, immune response regulation, and maintaining intestinal barrier integrity. *Akkermansia muciniphila*’s ability to decrease gene expression associated with adipocyte differentiation and lipid oxidation, suppress inflammation, and support lipid clearance mechanisms under metabolic stress suggests that it offers a protective effect against metabolic diseases. Additionally, it strengthens the intestinal barrier by increasing the number of goblet cells, thickening the mucus layer, and alleviating endotoxemia and systemic inflammation by increasing tight junction protein expression. These effects have also yielded beneficial results in colitis, liver inflammation, and atherosclerosis. Additionally, interactions between this bacterium and the immune system include the induction of regulatory T cells (T_reg_), the suppression of proinflammatory cytokines, and the regulation of TLR2 activation [43]. In an experimental Alzheimer’s model, oral administration of *Akkermansia muciniphila* has been reported to increase propionic acid levels in serum and cerebrospinal fluid, thereby suppressing the overactivation of mitochondrial fission protein 1 (DRP1). Signaling processes such as ubiquitination and phosphorylation, which are essential for mitochondrial autophagy (mitophagy), are regulated by the PINK1/PARKIN protein. The same study also found that *Akkermansia muciniphila* enhances mitophagy by increasing PINK1 expression. The normalization of mitophagy and the preservation of mitochondrial homeostasis via *Akkermansia muciniphila* is a remarkably significant finding [44].

Within this comprehensive neurobiological framework, the aim of the present study is to evaluate the beneficial effects of intermittent fasting and administration of *Akkermansia muciniphila* on behavioral, neuroinflammatory, apoptotic, autophagic, and GABAergic biomarkers in an autism-like phenotype induced by prenatal VPA exposure. In this context, molecular responses in the prefrontal cortex and hippocampus and associated behavioral outcomes were examined to compare the separate and combined effects of the two interventions. We hypothesized that intermittent fasting and *Akkermansia muciniphila* administration would improve behavioral abnormalities and key molecular pathways associated with autism, and that their combined application would produce broader effects than either intervention alone.

## 2. Materials and Methods

### 2.1. Animals and Experimental Design

Male offspring of Wistar rats exposed to VPA during pregnancy were used in this study. This choice was based on the well-established male bias in autism spectrum disorder prevalence and previous VPA-model studies showing more robust and reproducible behavioral and molecular phenotypes in male offspring [45]. In addition, the inclusion of both sexes would have substantially increased the sample size requirements and introduced sex as an additional biological variable, which was beyond the scope of the present study. All experimental procedures were carried out at the Erciyes University Experimental Studies Application and Research Center (DEKAM), and ethical approval for the study was obtained from the Erciyes University Animal Experiments Ethics Committee (Decision number: 24/083; Date: 8 May 2024). The animals were housed under standard room conditions throughout the experimental procedure, with a temperature of 23 ± 1 °C, a humidity level of 55–60%, and a 12-h light/dark cycle (illuminated between 7:00 a.m. and 7:00 p.m.). To breed the animals participating in the experiment, 15 females and 15 males were caged daily for 12 h (between 7:00 p.m. and 7:00 a.m.) at a 1:1 male-to-female ratio until spermatozoa were observed in the vaginal smear. Vaginal smears were collected from female animals at the end of each day of male–female caging and examined under a light microscope. The day that spermatozoa were observed in the vaginal smear was considered the first gestational day (gestational day 0.5 = G0.5), and the animal considered as pregnant was caged individually on that day. The most widely accepted dose and application day ensuring the construct and face validity of the VPA-induced autism model are 500 mg/kg and gestational day 12.5 (G12.5), respectively [45]. Therefore, in this study, 12 female animals considered pregnant were injected with 500 mg/kg of VPA (Sigma-Aldrich Chemical Co., St. Louis, MO, USA, Product no: P4543) intraperitoneally (i.p.) at G12.5 (dissolution volume 200 mg/mL) to establish the autism model, while 3 female animals considered pregnant were injected with the same volume of saline on the same day (G12.5). The study design required multiple treatment groups generated from VPA-exposed pups (VPA, IF, probiotic, IF + probiotic), whereas only one saline-exposed control group was needed. To ensure sufficient numbers of male offspring for multiple VPA-derived experimental groups while accounting for variability in the success of pregnancy, litter size, and sex distribution, 12 dams were assigned to the VPA condition and 3 dams to the saline control condition. This approach is consistent with common practice in prenatal VPA studies, where the majority of offspring are required from the VPA condition to populate several experimental arms [46]. The gestation period lasted 21–23 days and, at the end of this period, each rat gave birth to 7–12 pups. To prevent variability in the growth and development of the pups, some pups were culled on the first day after birth, ensuring that each mother (dam) nursed a maximum of 7 pups. Culling procedures were performed using a humane euthanasia method appropriate for neonatal rodents, in accordance with the AVMA Guidelines for the Euthanasia of Animals [47] and institutional ethical approval. Neonatal pups were euthanized by rapid decapitation using sharp surgical scissors, performed by trained personnel to ensure immediate loss of consciousness. All efforts were made to minimize distress and handling time during the procedure.

The sample size was determined based on previous studies using the prenatal VPA-induced model evaluating behavioral and molecular outcomes, where group sizes of 6–10 animals are commonly sufficient to detect biologically meaningful differences [48,49,50]. In addition, an a priori sensitivity analysis (G*Power 3.1; one-way ANOVA, five groups, α = 0.05, power (1 − β) = 0.80) indicated that a sample size of *n* = 8 per group was sufficient to detect medium-to-large effects. Considering the ethical principles of animal research (3R reduction principle) and feasibility constraints, a target sample size of *n* = 8 male offspring per group was defined. Thus, 32 male offspring born to dams exposed to VPA and 8 male offspring born to dams exposed to saline were included in this study. Importantly, for the VPA-exposed groups, offspring were randomly selected from different litters, and no more than two pups from the same litter were assigned to the same experimental group to minimize litter-specific bias and pseudo-replication and prevent the overrepresentation of pups from the same litter within a single experimental group. Accordingly, care was taken to ensure that each experimental group included offspring from at least four different dams, thereby minimizing the likelihood that observed group effects were driven by dam-specific biological variability. The allocation of offspring to experimental groups was performed using a computer-generated randomization procedure, and the group assignment was carried out by a researcher who was not involved in the behavioral testing or biochemical analyses to minimize allocation bias.

The lactation period lasted 21 days, and on postnatal day 22 (P22), the pups were separated from their mothers for assignment to the experimental groups. The treatment period lasted 30 days after the lactation period (between P22 and P51). The rats in the treatment groups were subjected to intermittent fasting and the next-generation probiotic *Akkermansia muciniphila* (AkkermansiaTR^®^, Istanbul, Turkey, Dietary supplement approval number: 015626-20.12.2022) intervention, either separately or in combination. The therapeutic dose for *Akkermansia muciniphila* was 1 × 10^9^ cfu/day, dissolved in 0.25 mL saline and administered via oral gavage. The intermittent fasting protocol was implemented as a time-restricted eating model [51]. The animals subjected to intermittent fasting were fasted for 16 h per day (4:00 p.m. to 8:00 a.m.) for 30 days, while they were allowed ad libitum feeding for 8 h (8:00 a.m. to 4:00 p.m.). They also had ad libitum access to water during the experiment.

The experimental groups were as follows:

Control group: Rats were exposed to a single dose of saline at G12.5. In addition, 0.25 mL/day of saline was administered via oral gavage during the treatment period.

Autistic-like (VPA) group: Rats were exposed to a single dose of VPA at G12.5. In addition, 0.25 mL/day saline was administered via oral gavage during the treatment period.

Intermittent fasting (IF) treatment group: Rats were exposed to a single dose of VPA at G12.5 and fasted for 16 h daily during the treatment period. In addition, 0.25 mL/day of saline was administered via oral gavage during the treatment period.

*Akkermansia muciniphila* treatment (probiotic) group: Rats were exposed to a single dose of VPA at G12.5. In addition, *Akkermansia muciniphila* (1 × 10^9^ cfu/day) was administered via oral gavage at a dose of 0.25 mL/day during the treatment period.

Intermittent fasting + *Akkermansia muciniphila* treatment (IF + probiotic) group: Rats were exposed to a single dose of VPA at G12.5. They were also fasted for 16 h daily and administered 0.25 mL/day of *Akkermansia muciniphila* supplementation (1 × 10^9^ cfu/day) via oral gavage during the treatment period.

### 2.2. Animal Welfare and Compliance with the 3Rs Principles

The experiments complied with ARRIVE guidelines and were performed in accordance with the UK Animals (Scientific Procedures) Act, 1986 and related guidelines, and EU Directive 2010/63/EU for animal testing. The 3Rs principles (Reduction, Refinement, and Replacement) were considered in the study design [52].

Reduction: The number of animals was determined by targeting the minimum sample size sufficient to obtain statistically significant results. Both behavioral and biochemical parameters were analyzed in the same animals, thus avoiding the use of additional animals. Moreover, offspring were selected from different mothers, and the number of siblings in the same group was limited.

Refinement: All procedures were performed to minimize stress and pain in the animals. The animals were housed under standard conditions, unnecessary manipulation was avoided, and all injections were performed by experienced personnel using sterile technique and appropriate needle size to minimize tissue trauma. Particular attention was paid to maternal welfare following high-dose VPA administration, given its known potential for transient toxicity. Pregnant dams were continuously observed for the first 2 h post-injection and monitored at least twice daily thereafter until parturition. Clinical monitoring included assessment of posture, locomotor activity, piloerection, grooming behavior, body weight changes, and respiratory signs or abdominal discomfort. A predefined humane endpoint protocol was established, including criteria such as body weight loss (>20%), severe lethargy, and persistent abnormal posture. Routine welfare checks were conducted daily throughout gestation and the intervention period. Given the implementation of a 16-h intermittent fasting protocol, particular attention was paid to welfare monitoring for young rats. They were observed daily for signs of dehydration, hypoactivity, abnormal posture, piloerection, and reduced grooming throughout the intervention period. Body weight was recorded at regular intervals (every four days) to monitor growth trajectories and detect potential adverse effects. No animals met humane endpoint criteria, and no mortality occurred. Additionally, neonatal culling procedures were conducted in strict accordance with humane euthanasia guidelines to minimize suffering.

Replacement: Since the scope of the study required the combined assessment of behavioral symptoms and brain biomarkers, alternative in vitro methods were deemed insufficient, and the use of an in vivo model was considered necessary.

### 2.3. Behavioral Tests

Behavioral tests were conducted during the last eight days of the treatment period (P44–P51). To investigate the potential therapeutic effects of IF and *Akkermansia muciniphila* supplementation on VPA-induced autism-like symptoms, we examined five key behavioral patterns corresponding to behavioral domains frequently altered in autism: Stereotypy, visual recognition memory, social interaction, spatial memory, and anhedonia. These behavioral domains were assessed via repetitive self-grooming behavior (P44), novel object recognition (P44–P45), three-chamber social interaction (P46–P47), Y-maze (P48), and sucrose preference tests (P49–P51), respectively. All behavioral tests were conducted between 11:00 a.m. and 04:00 p.m.

#### 2.3.1. Repetitive Self-Grooming Behavior Test

Stereotypy, one of the core symptoms of autism, manifests itself through behaviors such as self-grooming, digging, circling, and jumping. In the present study, self-grooming behavior (face rubbing and paw/fur licking) was assessed in open field conditions in accordance with standard protocols used in previous animal studies [53]. For the test, each rat was placed in the center of an open-ceiling plexiglass apparatus measuring 100 cm × 100 cm × 40 cm, and their behavior was recorded by a video camera for 10 min. Between each trial, the inner surfaces of the apparatus were cleaned with 70% ethanol and thoroughly ventilated. This prevented odor-based orientation. All analyses were manually scored by a researcher blinded to groups. This methodological approach is a valid and reproducible measurement technique that allows for the reliable quantification of stereotypical behaviors [53].

#### 2.3.2. Novel Object Recognition Test

The novel object recognition test is a method commonly used to assess visual memory and identify cognitive impairments. The test protocol is based on the standardized application developed by Bevins and Besheer [54]. For this purpose, an open-ceiling plexiglass apparatus measuring 50 cm × 50 cm × 40 cm was used. The test was conducted over two days: a habituation day and a test day. On the habituation day, the animals were allowed to become accustomed to the test apparatus for 60 min: no objects were placed in the apparatus during this phase. On the test day, a two-stage procedure was carried out: the familiarization trial and the test trial. In the familiarization trial, two identical objects were placed in opposite corners of the apparatus (10 cm distant from each wall). The test animal was placed in the center and allowed to explore these objects for 3 min. In the test phase, conducted after a one-hour interval, one of these objects was replaced with a novel object, and the test animal was placed back in the apparatus. During the test phase, the animal’s behavior was recorded with a video camera for 3 min. Sniffing behavior at a distance of less than 2 cm from the objects was considered exploration, and all evaluations were performed manually by a blinded researcher. Between each trial, the apparatus and objects were cleaned with 70% ethanol and thoroughly ventilated. Cognitive performance was calculated using the discrimination index (DI), which reflects the animal’s preference level for the novel object compared to the former object.

$$DI=\frac{Exploration\;time\;of\;novel\;object\;\left(s\right)-Exploration\;time\;of\;former\;object}{Exploration\;time\;of\;novel\;object\;\left(s\right)+Exploration\;time\;of\;former\;object}$$

#### 2.3.3. Three-Chamber Social Interaction Test

Another hallmark of autism involves deficits in social interaction, reflected in rodents by diminished engagement or reluctance to interact with unfamiliar animals. The three-chamber social interaction test is a standard method widely used to assess sociability and social novelty preference in rodents and has high validity in identifying autism-like social behavior disorders [55]. The test apparatus consists of three open-ceiling plexiglass chambers, each measuring 40 cm × 40 cm × 40 cm, placed side by side and connected to each other by sliding doors [56]. The protocol consists of four phases: two habituation phases, a sociability task, and a social preference task. The first habituation phase was performed on the first day, and the other phases were performed on the second day. In the first habituation phase, the test animal was allowed to freely roam in the apparatus for 15 min to become accustomed to the environment. The second habituation phase, performed the following day (24 h after the first habituation), was a brief 10-min reminder session. In the sociability task, a cage containing an unfamiliar rat (stranger rat-1) of the same age and sex was placed in the right chamber, while an empty cage was placed in the left chamber. The test animal was placed in the middle chamber. After the sliding doors were opened, the test animal’s behavior was recorded by a video camera for 10 min. Upon completion of this phase, the test animal was returned to the waiting position in the middle chamber, and the sliding doors were closed. Subsequently, the position of the cages was changed for the social preference phase, and a new unfamiliar rat (stranger rat-2) was placed in the empty cage. The test animal was prevented from observing these changes. The sliding doors were reopened to initiate the 10-min social preference task, and the test animal’s behavior was recorded by a video camera during this task as well. In both tasks, interest behaviors (sniffing, climbing, and pacing) directed toward the unfamiliar animals/empty cage were measured based on duration, and scoring was performed manually by a researcher blinded to groups. Between all trials, the chambers and cages were cleaned with 70% ethanol to prevent possible odor-based orientation. The parameter assessed in the sociability task was the “sociability index (SI)”, while the parameter assessed in the social preference task was the “social preference index (SPI)”. SI and SPI were calculated as follows:

$$\;SI=\frac{Time\;spent\;with\;stranger\;rat-1\;(s)}{Time\;spent\;with\;empty\;cage\;(s)}$$

$$SPI=\frac{Time\;spent\;with\;stranger\;rat-2\;(s)}{Time\;spent\;with\;stranger\;rat-1\;(s)}$$

#### 2.3.4. Y-Maze Test

Animals tend to alternate between different options, which requires the ability to remember the previous choice. The Y-maze spontaneous alternation test is a simple yet highly valid behavioral test that allows for the assessment of short-term spatial memory function in rodents [57]. The test apparatus consists of an open-ceiling maze with three equal arms connected at 120° angles. The protocol consists of two phases conducted 24 h apart: habituation and test phases. During the habituation phase, the test animal was placed in the maze and allowed to explore freely for 5 min. On the test day, the animal is placed back in the center of the maze and all arm entries are recorded by a video camera for 5 min. An alternation is defined as the animal entering three different arms in sequence (e.g., ABC, BCA, CAB, ABC, BCA). To prevent odor-based orientation, all inner surfaces of the maze were cleaned with 70% ethanol and thoroughly ventilated between each trial. The spontaneous alternation rate was calculated by dividing the total number of alternations by the total number of arm entries minus two [58]:

$$\;Spontaneous\;alternation\;(\%)=\frac{Number\;of\;total\;alternations}{Number\;of\;total\;arm\;visits-2}\times 100$$

#### 2.3.5. Sucrose Preference Test

Rodents tend to prefer sweet foods and beverages over bland ones as an indicator of hedonic behavior. Less preference for sweet foods and beverages reflects anhedonia. The sucrose preference test is a reward-based behavioral assessment method used to evaluate anhedonic behavior. The test protocol reported by Liu et al. [59] was used in this study. For pre-test acclimatization, two water bottles (both containing only water) were placed in all animal cages for three days, and the daily consumption difference between the bottles was measured to confirm whether there was any bias in preference. During the test phase, each animal was placed individually in a cage and given free access to two bottles for 12 h, one containing a 1% sucrose solution and the other containing water. The test was conducted between 7:00 p.m. and 7:00 a.m. because rodents’ drinking behavior is more pronounced at night. After this initial 12-h consumption period, the animals were deprived of water and food for 24 h. Subsequently, access to the two bottles (1% sucrose solution and water) was provided again for 12 h. The volumes of sucrose solution and water consumed during the final 12-h period were recorded, and the sucrose preference percentage was calculated using the following formula:

$$Sucrose\;preference\;(\%)=\frac{Sucrose\;solution\;intake}{Sucrose\;solution\;intake+Water\;intake}\times 100$$

### 2.4. Blood and Brain Tissue Collection

Following behavioral tests, animals were euthanized under anesthesia according to experimental protocols using a combination of ketamine (80 mg/kg) and xylazine (10 mg/kg). Maximum cardiac blood was collected when the anesthetic depth was reached at which the nociceptive flexion reflex was lost. Immediately afterwards, the rats were decapitated, brain tissues were quickly removed from the skulls, and the prefrontal cortex and ventral hippocampus were dissected. Meanwhile, the blood was centrifuged at 3000 rpm and 4 °C for 25 min to separate the serum. Serum samples and dissected tissues were rapidly shock-frozen with liquid nitrogen and stored at −80 °C until biochemical analysis.

### 2.5. Biochemical Analyses

The serum (zonulin) and brain tissue (IL-1β, IL-6, TNF-α, IFN-γ, BAX, BCL-2, LC3-II, Beclin-1, glutamate decarboxylase-67 (GAD67), and parvalbumin) parameters were measured using the enzyme-linked immunosorbent assay (ELISA) method with commercial ELISA kits in accordance with the manufacturer’s protocol (Shanghai Sunred Biological Technology Co., Shanghai, China; Catalog numbers: 201-11-0120 (IL-1β), 201-11-0136 (IL-6), 201-11-0765 (TNF-α), 201-11-0104 (IFN-γ), 201-11-0035 (BAX), 201-11-0038 (BCL-2), 201-11-1701 (LC3-II), 201-11-1689 (Beclin-1), 201-11-2306 (GAD67), 201-11-1444 (parvalbumin), and 201-11-5578 (zonulin)). For all biochemical analyses, each animal was considered a biological replicate. Serum and brain tissue samples obtained from each rat were analyzed separately without pooling. All ELISA measurements were technically duplicated, and the mean of the two wells was used for statistical analysis. Samples were randomly distributed across plates and analyzed within the same assay run whenever possible to reduce batch effects. The intra- and inter-assay coefficients of variation (CVs) provided by the manufacturer were <10% and <12%, respectively, and all measurements in this study were performed within these recommended ranges. Measurements with CV > 15% were repeated. The researchers that performed the ELISA analyses were blinded to the group allocation.

Direct concentration values were recorded for serum samples while, for brain tissues, the concentration values of the parameters were divided by the protein concentration to standardize them, and the values per unit amount of protein were calculated. A homogenization procedure was performed before analyzing the brain tissue parameters: first, 50 mg of tissue was homogenized in 500 μL (0.1 M) phosphate-buffered saline solution (pH: 7.4) for 5 min using an ultrasonic homogenizer. Subsequently, the homogenized tissues were centrifuged at 10,000 rpm and 4 °C for 15 min, and the supernatants were transferred to Eppendorf tubes and stored at −80 °C until the day of analysis. A commercial bicinchoninic acid (BCA) protein assay kit was used to determine the total protein concentrations in the tissues (Thermo Fisher Scientific Inc., Waltham, MA, USA, Catalog number: A65453).

**Figure 1:**
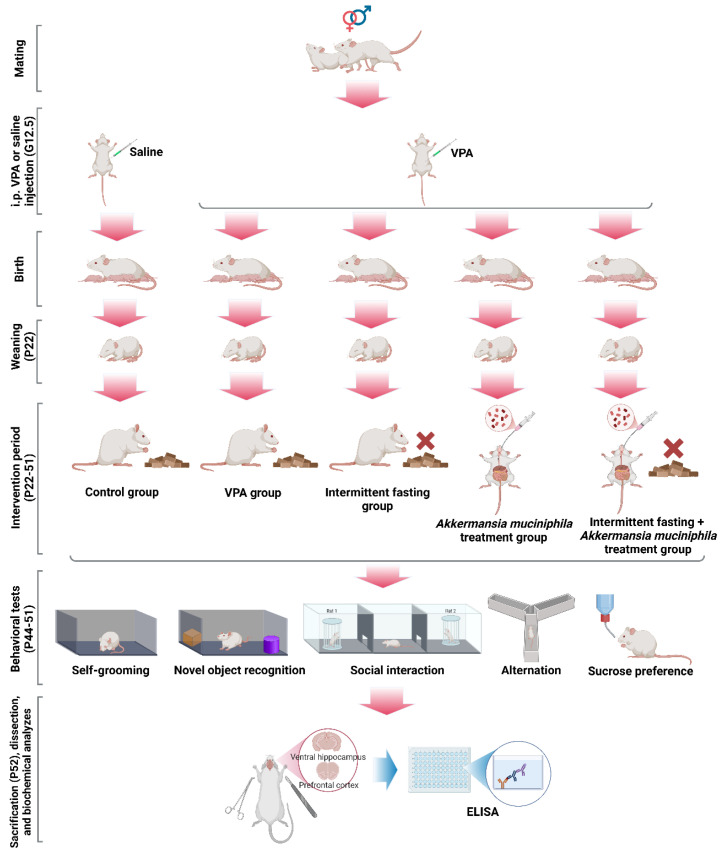
Overview of study methodology. Rats detected to be mated were injected with VPA or saline i.p. at G12.5. Offspring exposed to saline during the prenatal period were assigned to the control group, while those exposed to VPA were randomly assigned to the VPA or treatment groups. All offspring were allowed to be breastfed. The treatments were administered for 30 days following the lactation period. Behavioral tests were performed during the last eight days of the treatment period. Finally, all animals were euthanized after the treatment period.

A methodological overview of the study is presented in Figure 1.

### 2.6. Statistical Analyses

In accordance with ARRIVE 2.0 guidelines, the primary outcome of the study was pre-specified as the sociability index (SI) derived from the three-chamber social interaction test, as deficits in social interaction represent a core behavioral domain in autism. The sample size determination and sensitivity analysis were primarily based on detecting biologically meaningful differences in this endpoint. All other behavioral and biochemical parameters were considered secondary outcomes and were interpreted as exploratory endpoints.

All statistical analyses were performed using the GraphPad Prism statistical graphics software (version 10.2.0; GraphPad Software Inc., San Diego, CA, USA). Data are presented as means and standard errors (SE) in bar graphs. The normality of the data was assessed using the Shapiro–Wilk test. Body weight was monitored every four days throughout the intervention period and analyzed using two-way repeated measures ANOVA (group × time) to evaluate differences in growth trajectories between groups. For all other behavioral and biochemical parameters, one-way ANOVA was used to detect statistical differences between groups. The VPA group was used as the reference group for comparisons. All graphs show whether the control and treatment groups differed from the VPA group. Tukey’s multiple comparison test was preferred as a post hoc method to determine statistically different groups, and type 1 error was accepted as *p* < 0.05. In addition, effect sizes were calculated to facilitate interpretation of the magnitude of the group differences. Partial eta squared (η^2^p) is reported for one-way ANOVA as a measure of the overall effect size, and Cohen’s d was calculated for post hoc pairwise comparisons relative to the VPA group.

## 3. Results

**Figure 2:**
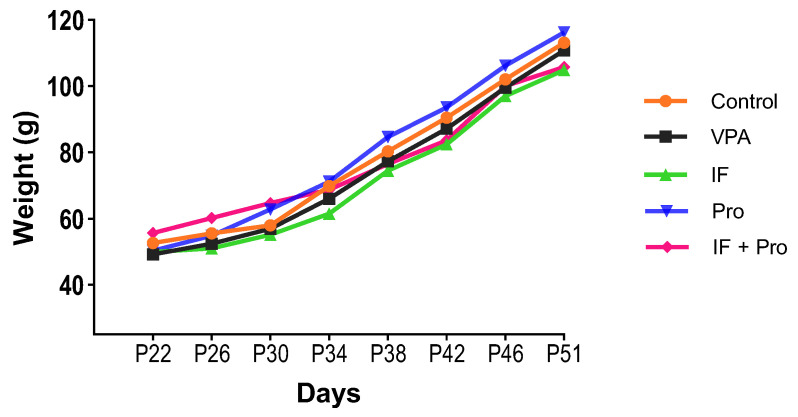
Comparison of the groups in terms of body weight trajectories. The differences in baseline body weight between groups were not significant. In addition, no significant group × time interaction effect on body weight development was detected during the intervention period. Data are presented as mean values. *n* = 8 rats per group.

Body weight measurements obtained every four days during the intervention period are presented in Figure 2. There were no significant differences in baseline body weight between groups (*p* > 0.05). A significant increase in body weight was observed in all groups over time (*p* < 0.001). However, two-way repeated measures ANOVA did not reveal a significant group × time interaction effect on body weight development during the intervention period (*p* > 0.05).

**Figure 3:**
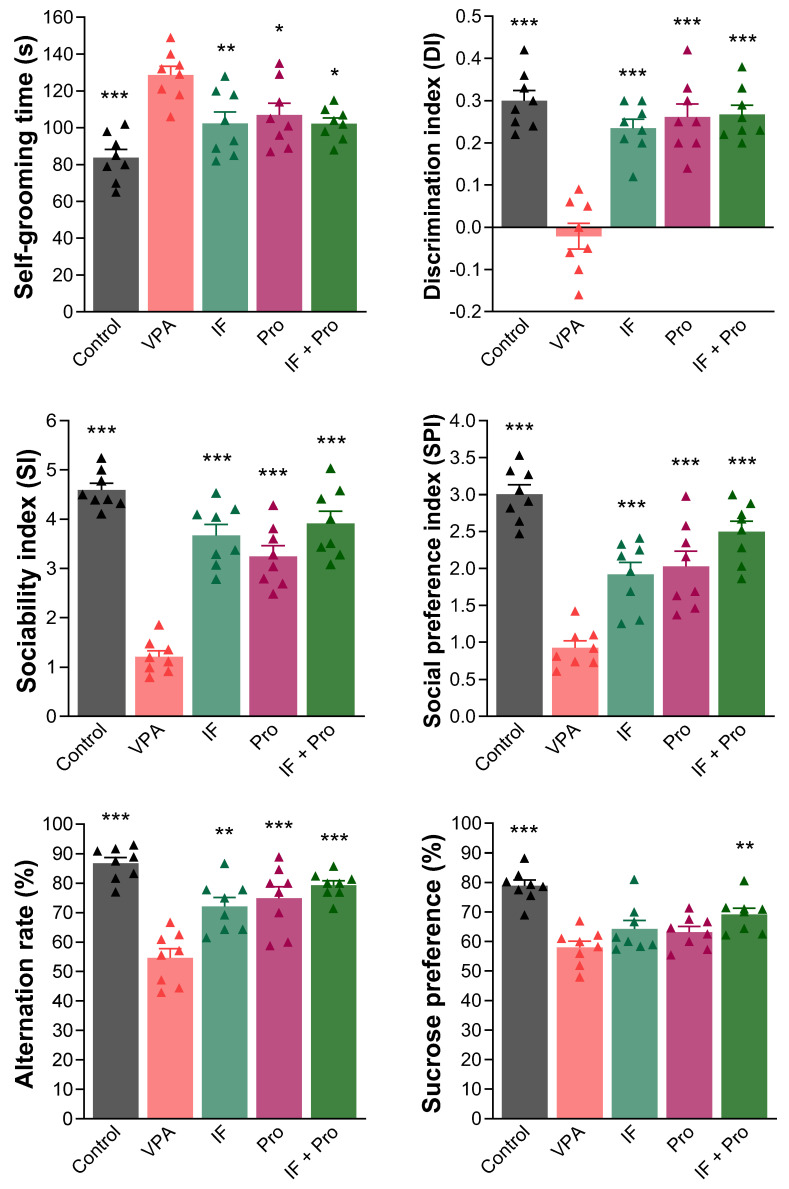
Comparison of the groups in terms of the behavioral parameters. Prenatal VPA exposure worsened all behavioral outcomes. In contrast, the self-grooming time, DI, SI, and SPI scores, and the alternation rates were significantly higher in all treatment groups than in the VPA group. In addition, the only group that differed from the VPA group in terms of the sucrose preference was the IF + probiotic combination treatment group. Data are presented as mean and SE. *n* = 8 rats per group. * *p* < 0.05, ** *p* < 0.01, and *** *p* < 0.001 compared to the VPA group.

One-way ANOVA revealed large overall effects for behavioral outcomes, with partial η^2^ values of 0.527 for self-grooming, 0.831 for SI, 0.750 for SPI, 0.742 for DI, 0.666 for the alternation rate, and 0.590 for sucrose preference. Pairwise comparisons relative to the VPA group showed large effect sizes across tests, with Cohen’s d values ranging from 1.37 to 3.40 for the self-grooming time, 4.09–9.35 for SI, 2.45–6.59 for SPI, 3.24–4.15 for DI, 1.98–4.33 for the alternation rate, and 0.88–3.63 for sucrose preference. The results of the behavioral tests are presented in Figure 3. Prenatal VPA administration triggered behavioral symptoms such as stereotypy (increased self-grooming), social impairment (decreased SI and SPI), poor object memory (decreased DI), spatial memory deficit (decreased alternation rate), and anhedonia (decreased sucrose preference) (*p* < 0.001). The treatments generally reversed VPA-induced behavioral symptoms, and all three treatments reduced self-grooming times; increased DI, SI, and SPI scores; and improved alternation rates (*p* < 0.05). The IF-only treatment was found to be more effective than the probiotic-only and IF + probiotic treatments in reducing self-grooming times but less effective at improving alternation rates. In contrast, the sucrose preference ratios did not improve with the IF-only and probiotic-only treatments (*p* > 0.05). An increase in sucrose preference was observed with the IF + probiotic combination treatment (*p* < 0.01).

**Figure 4:**
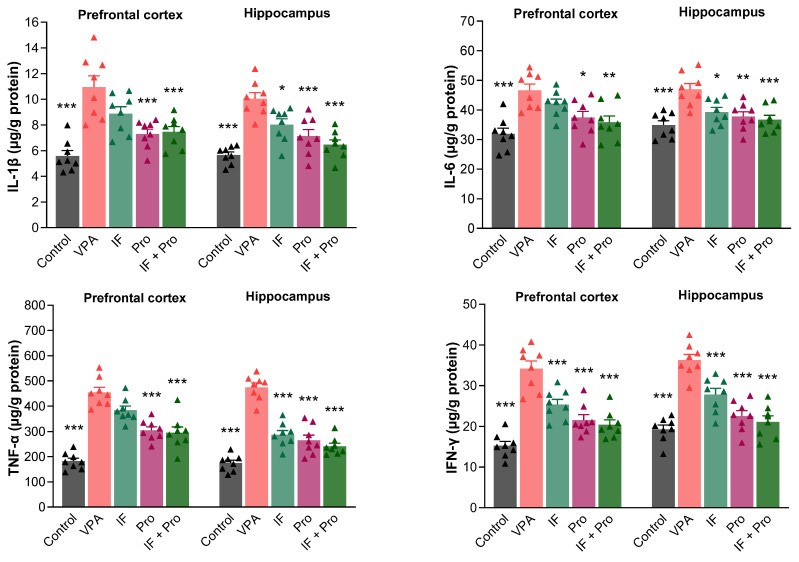
Comparison of the groups in terms of the proinflammatory mediator levels in the prefrontal cortex and hippocampus. Prenatal VPA exposure increased all inflammatory cytokine levels in both prefrontal cortex and hippocampus. All treatments were successful at decreasing the hippocampal inflammatory cytokine levels. On the other hand, in the prefrontal cortex, the probiotic-only and IF + probiotic treatments showed anti-inflammatory effects, while the IF-only treatment did not significantly affect IL-1β, IL-6, and TNF-α levels. Data are presented as mean and SE. *n* = 8 rats per group. * *p* < 0.05, ** *p* < 0.01, and *** *p* < 0.001 compared to the VPA group.

One-way ANOVA also revealed large overall effects for neuroinflammatory markers. In the prefrontal cortex, the partial η^2^ values were 0.596 for IL-1β, 0.491 for IL-6, 0.795 for TNF-α, and 0.755 for IFN-γ, while in the hippocampus they were 0.643, 0.482, 0.849, and 0.749, respectively. Pairwise comparisons relative to the VPA group also showed large effect sizes, with Cohen’s d values ranging from 1.01 to 4.14 for IL-1β, from 1.53 to 8.86 for IL-6, from 1.38 to 7.22 for TNF-α, and from 0.81 to 4.87 for IFN-γ across the prefrontal cortex and hippocampus. The levels of proinflammatory mediators (IL-1β, IL-6, TNF-α, and IFN-γ) in the prefrontal cortex and hippocampus of rats are presented in Figure 4. Prenatal VPA exposure increased the levels of these inflammatory cytokines in both the prefrontal cortex and hippocampus (*p* < 0.001). The IF-only treatment did not significantly improve the inflammatory cytokine levels, other than that of IFN-γ in the prefrontal cortex (*p* > 0.05); in contrast, the probiotic-only and IF + probiotic treatments reduced all inflammatory cytokine levels in the prefrontal cortex (*p* < 0.05). Moreover, the hippocampal inflammatory cytokine levels were attenuated by all treatments (*p* < 0.05). The treatments were ranked in terms of their anti-inflammatory effects in hippocampal tissue as follows: IF + probiotic > probiotic > IF.

**Figure 5:**
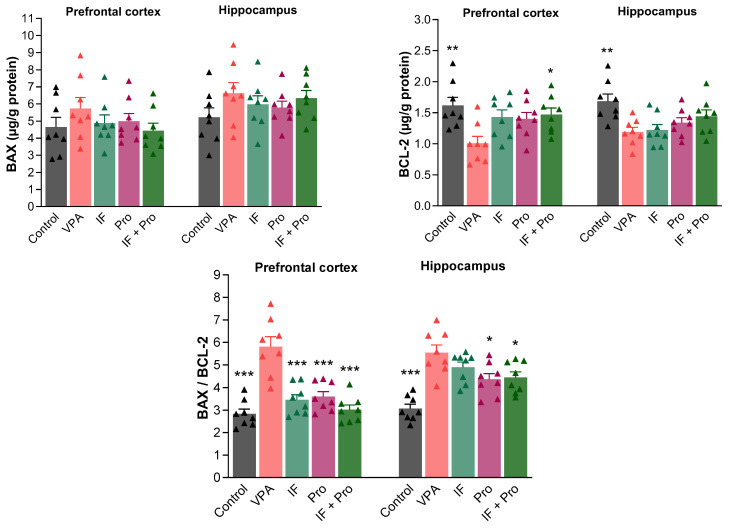
Comparison of the groups in terms of the BAX and BCL-2 levels and BAX/BCL-2 ratios in the prefrontal cortex and hippocampus. No significant difference in the BAX levels was observed between groups. However, prenatal VPA exposure decreased the BCL-2 levels and increased the BAX/BCL-2 ratios in both the prefrontal cortex and hippocampus. While none of the treatments were successful in increasing the hippocampal BCL-2 level, the IF + probiotic treatment increased the prefrontal cortex BCL-2 level. In addition, both probiotic-only and IF + probiotic treatments decreased the BAX/BCL-2 ratios in both the prefrontal cortex and hippocampus. The IF-only treatment, on the other hand, only decreased the BAX/BCL-2 ratios in the prefrontal cortex. Data are presented as mean and SE. *n* = 8 rats per group. * *p* < 0.05, ** *p* < 0.01, and *** *p* < 0.001 compared to the VPA group.

One-way ANOVA revealed variable overall effects for apoptosis-related markers. In the prefrontal cortex, the partial η^2^ values were 0.094 for BAX, 0.312 for BCL-2, and 0.678 for the BAX/BCL-2 ratio, while in the hippocampus they were 0.115, 0.345, and 0.589, respectively. Pairwise comparisons relative to the VPA group showed small to very large effect sizes, with Cohen’s d values ranging from 0.19 to 0.85 for BAX, from 0.14 to 1.83 for BCL-2, and from 0.80 to 3.18 for the BAX/BCL-2 ratio across the prefrontal cortex and hippocampus. The BAX and BCL-2 levels and BAX/BCL-2 ratios in the prefrontal cortex and hippocampus of rats are shown in Figure 5. Prenatal VPA administration increased the BAX levels in both the prefrontal cortex and hippocampus (*p* < 0.01). No significant difference was found between groups in terms of the BAX levels in both the prefrontal cortex and hippocampus (*p* > 0.05). In contrast, prenatal VPA administration decreased the BCL-2 levels in the prefrontal cortex and hippocampus (*p* < 0.01). The IF + probiotic combined treatment was effective at increasing the prefrontal cortex BCL-2 level (*p* < 0.05). However, no treatment significantly increased the hippocampal BCL-2 level (*p* > 0.05). Differences between groups were more apparent in terms of the BAX/BCL-2 ratios. Prenatal VPA administration increased the BAX/BCL-2 ratios in both the prefrontal cortex and hippocampus (*p* < 0.001), and all treatments decreased the BAX/BCL-2 ratios in the prefrontal cortex (*p* < 0.001). Moreover, the probiotic-only and IF + probiotic treatments were successful in reducing the hippocampal BAX/BCL-2 ratios (*p* < 0.05), while the IF-only treatment did not improve these ratios (*p* > 0.05).

**Figure 6:**
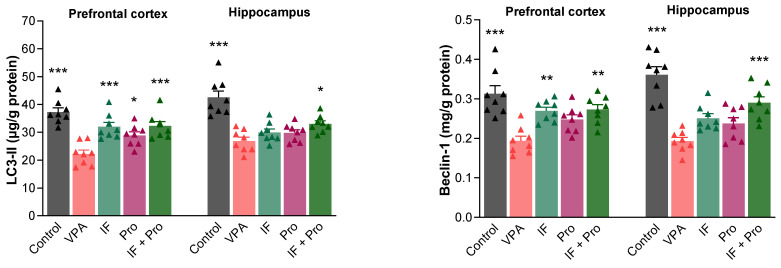
Comparison of the groups in terms of the LC3-II and Beclin-1 levels in the prefrontal cortex and hippocampus. Prenatal VPA exposure decreased the LC3-II and Beclin-1 levels in both the prefrontal cortex and hippocampus. In contrast, the IF + probiotic combination treatment was successful at improving the LC3-II and Beclin-1 levels in both brain regions. However, the probiotic-only treatment increased only prefrontal cortex LC3-II levels, while the IF-only treatment improved prefrontal LC3-II and Beclin-1 levels. Data are presented as mean and SE. *n* = 8 rats per group. * *p* < 0.05, ** *p* < 0.01, and *** *p* < 0.001 compared to the VPA group.

One-way ANOVA also revealed large overall effects for autophagy-related markers. In the prefrontal cortex, the partial η^2^ values were 0.612 for LC3-II and 0.535 for Beclin-1, while in the hippocampus they were 0.650 and 0.679, respectively. Pairwise comparisons relative to the VPA group showed large to very large effect sizes, with Cohen’s d values ranging from 0.81 to 3.63 for LC3-II and from 1.29 to 3.72 for Beclin-1 across the prefrontal cortex and hippocampus. The LC3-II and Beclin-1 levels in the prefrontal cortex and hippocampus of rats are shown in Figure 6. Prenatal VPA exposure reduced the LC3-II and Beclin-1 levels in both the prefrontal cortex and hippocampus (*p* < 0.001). Although all treatments reversed the prefrontal cortex LC3-II levels, only the IF + probiotic combined treatment improved the hippocampal LC3-II levels (*p* < 0.05). Meanwhile, the prefrontal cortex Beclin-1 levels improved with the IF-only and IF + probiotic treatments (*p* < 0.01) but were unaffected by the probiotic-only treatment (*p* > 0.05), while the VPA-induced decrease in hippocampal Beclin-1 was reversed only by the IF + probiotic combined treatment (*p* < 0.001).

**Figure 7:**
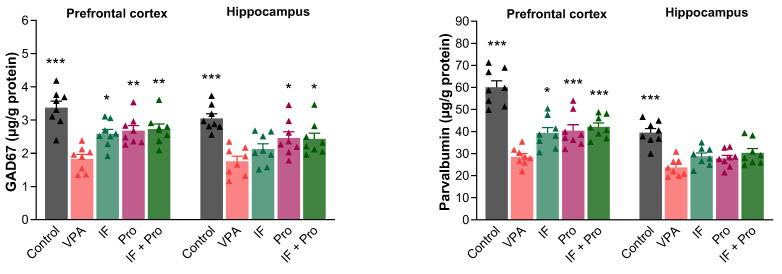
Comparison of the groups in terms of the GAD67 and parvalbumin levels in the prefrontal cortex and hippocampus. Prenatal VPA exposure decreased the GAD67 and parvalbumin levels in both the prefrontal cortex and hippocampus. In contrast, all treatments increased the GAD67 and parvalbumin levels in the prefrontal cortex. The hippocampal GAD67 levels were improved by the probiotic-only and IF + probiotic treatments, while none of the treatments significantly affected the hippocampal parvalbumin levels. Data are presented as mean and SE. *n* = 8 rats per group. * *p* < 0.05, ** *p* < 0.01, and *** *p* < 0.001 compared to the VPA group.

One-way ANOVA also revealed large overall effects for GABAergic markers. In the prefrontal cortex, partial η^2^ values were 0.585 for GAD67 and 0.731 for parvalbumin, while in the hippocampus they were 0.490 and 0.586, respectively. Pairwise comparisons relative to the VPA group showed large to very large effect sizes, with Cohen’s d values (vs. VPA) ranging from 0.82 to 3.26 for GAD67 and from 1.04 to 4.87 for parvalbumin across the prefrontal cortex and hippocampus. The GAD67 and parvalbumin levels in the prefrontal cortex and hippocampus of rats are presented in Figure 7. Prenatal VPA exposure was found to decrease the GAD67 and parvalbumin levels in both the prefrontal cortex and hippocampus (*p* < 0.001), while all three treatments reversed VPA-induced decreases in GAD67 and parvalbumin in the prefrontal cortex (*p* < 0.05). However, none of the treatments improved the hippocampal parvalbumin levels (*p* > 0.05). The hippocampal GAD67 levels increased with the probiotic-only and IF + probiotic treatments (*p* < 0.05) but were not affected by the IF-only treatment (*p* > 0.05).

**Figure 8:**
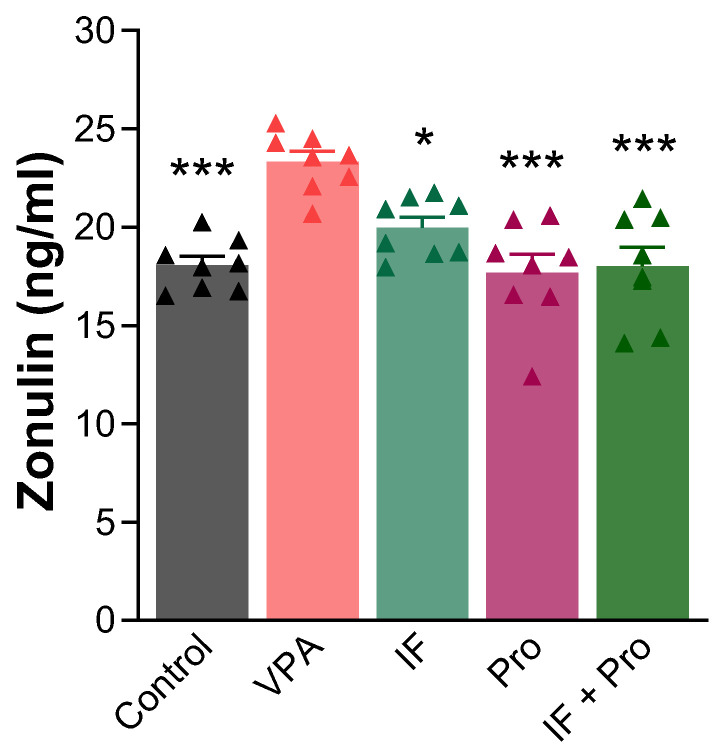
Comparison of the groups in terms of the serum zonulin levels. Prenatal VPA exposure increased the serum zonulin levels, whereas all treatments showed ameliorative effects. Data are presented as mean and SE. *n* = 8 rats per group. * *p* < 0.05 and *** *p* < 0.001 compared to the VPA group.

Lastly, a large overall effect was also observed for the serum zonulin levels (partial η^2^ = 0.554). Pairwise comparisons relative to the VPA group showed very large effect sizes, with Cohen’s d values ranging from 2.25 to 3.76. The serum zonulin levels of the groups are shown in Figure 8. Prenatal VPA exposure increased the serum zonulin levels (*p* < 0.001), which all treatments successfully reversed (*p* < 0.05).

## 4. Discussion

The lack of a standard pharmacological treatment that directly targets the core symptoms of autism, the prevalence of which is increasing every year, represents a significant clinical gap. Existing treatment approaches are largely based on lifelong rehabilitation models, particularly cognitive behavioral therapy, applied behavior analysis, and social skills training. However, while these methods may improve functionality, they may not be sufficiently effective in addressing all symptom clusters. For this reason, complementary and supportive treatment options have increasingly become alternatives to reduce the severity of behavioral and neurobiological symptoms associated with autism. In recent years, certain dietary approaches and supplements have attracted therapeutic interest and it has been suggested that these complementary approaches may alleviate autism-specific behavioral and biochemical impairments through mechanisms such as reducing inflammation, synaptic plasticity effects, and modulating neuroactive metabolite production. There has been a strong focus on the effects of dietary models such as the gluten-free/casein-free diet, ketogenic diet, specific carbohydrate diet, and low-oxalate diet on symptoms associated with autism. However, it has been observed that the symptom-alleviating effects of these dietary approaches are limited [60,61,62,63]. Recent scientific interest has focused on the possibility that intermittent fasting may affect brain activity through metabolic, cellular, and circadian mechanisms [64]. The molecular mechanisms underlying the modulatory effect of intermittent fasting on autophagic flux are based on the opposite actions between AMPK and mTOR and lysosomal activities. An increase in the cellular AMP/ATP ratio under energy deprivation conditions leads to the activation of the energy sensor AMPK, which triggers autophagy by directly phosphorylating the ULK1 complex and simultaneously suppresses mTORC1—the negative regulator of anabolic processes—demonstrating that autophagy is one of the fundamental adaptive mechanisms against energy stress. mTORC1 is the primary inhibitor that remains active during periods of energy and nutrient abundance, thereby suppressing autophagy. The opposing effects of AMPK (which supports catabolism) and mTORC1 (which promotes anabolic processes) combined with the degradation capacity of lysosomes, enable the cell to adapt to environmental conditions [65]. Due to this mechanism that clears waste proteins from neurons, intermittent fasting is thought to contribute to symptom relief and slow disease progression in neurodegenerative and neuropsychiatric diseases [64]. The discovery that the next-generation probiotic *Akkermansia muciniphila*—which regulates energy metabolism, suppresses inflammation, and supports the immune response—enhances mitophagy by increasing PINK1 expression [44], suggests that it may have neuromodulatory potential. Therefore, experimentally testing the effectiveness of metabolic- and microbiota-targeted approaches, such as intermittent fasting and *Akkermansia muciniphila* supplementation, in targeting the multidimensional biological mechanisms of autism can be considered as an important area of research.

Within the scope of our study, it was determined that behavioral symptoms were generally impaired in the autism model. Prenatal VPA exposure reproduced a typical autism-like phenotype characterized by increased self-grooming behavior, impaired object recognition memory, decreased social interaction, declined short-term spatial memory performance, and the development of anhedonia. The fact that these VPA-induced behavioral abnormalities have been reported numerous times in the literature demonstrates that our current findings support the validity of the model [38,45,66,67,68]. Both intermittent fasting and *Akkermansia muciniphila* interventions, applied either separately or in combination, decreased the self-grooming intensity, improved object memory, and enhanced short-term spatial memory in VPA-exposed rats. Although the sucrose preference ratio did not increase with either intermittent fasting or *Akkermansia muciniphila* treatment, the combination of these two interventions significantly improved the hedonic response. Importantly, as the pre-specified primary outcome of the study, sociability performance (SI) was consistently improved across treatment groups, reinforcing the robustness and translational relevance of the observed behavioral effects. Findings reported in the literature regarding the effects of intermittent fasting on autism-related behavioral and molecular changes are quite limited. García-Juárez et al. [69] found that 5 weeks of alternate-day fasting improved autism-like social interaction deficits induced by a Western-style cafeteria diet. In another study, it was found that 60 days of alternate-day fasting in a *PTEN* neuronal haploinsufficiency model associated with the autism phenotype improved contextual fear memory impairment and decreased anxiety [70]. In addition, the potential of probiotic interventions to improve behavioral symptoms associated with autism has been investigated more extensively in both experimental and clinical studies. In our previous study, we demonstrated that VSL#3, a well-known multistrain probiotic containing *Lactobacillus*, *Bifidobacterium*, and *Lactococcus* species, administered at a dose of 22.5 × 10^9^ cfu/day for 6 weeks, reversed VPA-induced social interaction deficits, anxiety, and spatial memory impairment [38]. Mintál et al. [39] also reported that treatment with a probiotic supplement containing certain *Lactobacillus* and *Bifidobacterium* species for 2 weeks improved social interaction. Additionally, Kong et al. [71] compared three different *Lactobacillus* strains (at a dose of 1 × 10^9^ cfu/day for 4 weeks) in their research and noted that some strains reversed VPA-induced social impairment, stereotypy, and anxiety. On the other hand, findings from human studies are more heterogeneous, and these differences are thought to stem from limited sample sizes, different types of interventions, varying durations, and diversity in assessment tools. Some studies have indicated that probiotic intervention can rebalance sensory and behavioral symptoms, as well as the levels of neurotransmitters such as serotonin and dopamine, which play a critical role in behavior regulation [72,73,74,75], while other studies have emphasized that although probiotics have the potential to improve gastrointestinal symptoms, they do not significantly alleviate behavioral symptoms [76,77]. Taken together, the findings suggest that microbiota-targeted interventions are associated with improvements in autism-like behavioral symptoms, although the precise underlying mechanisms remain to be clarified. In particular, the fact that the effect of *Akkermansia muciniphila*, defined as a next-generation probiotic, on autism-related symptoms was demonstrated for the first time in this study, and that intermittent fasting—which has been studied in a limited number of cases in the existing literature—provided significant behavioral gains highlights the unique and innovative contributions of these two approaches. Furthermore, no significant differences in body weight trajectories were observed between groups during the intervention period, suggesting that the behavioral changes were not accompanied by differential growth patterns. Therefore, a more detailed elucidation of the biological mechanisms underlying these interventions is an important requirement for future research.

Evidence accumulated in recent years points to a significant link between inflammatory processes and autism. It has been shown that disruptions in microglial activity during brain development interfere with synaptic maturation [78,79]. These findings have paved the way for new explanatory hypotheses regarding the neurobiological basis of autism [80,81]. Studies conducted with animal models also strongly support the idea that proinflammatory cytokines play a direct role in the pathogenesis of autism [82,83]. In this context, the present study compared the levels of IL-1β, IL-6, TNF-α, and IFN-γ in the prefrontal cortex and hippocampus between groups. These two brain regions, which are central to neurodevelopmental processes associated with autism, are linked to distinct cognitive and social functions. The prefrontal cortex plays a critical role in decision making, language skills, and the execution of social and emotional processes and is directly associated with social interaction and communication difficulties, which are core symptoms of autism [84,85]. Meanwhile, the hippocampus plays a key role in learning and spatial memory processes, and it has been shown that the memory impairments frequently observed in autism are associated with functional changes occurring in this region [86]. Our results demonstrated that prenatal VPA exposure significantly increased the relevant proinflammatory cytokine levels in both the prefrontal cortex and hippocampus. Similarly, experimental studies using autism models have indicated increased inflammatory markers in various brain areas, including the prefrontal cortex and hippocampus [87,88,89,90]. In this study, we observed that all treatments were successful, particularly in alleviating hippocampal inflammation. Supporting our findings, various studies have indicated that intermittent fasting has mitigating effects on the neuroinflammatory response; for example, it has been shown that 30 days of alternate-day fasting can suppress increases in lipopolysaccharide-induced hippocampal IL-1α, IL-1β, and TNF-α levels [91]. Another study reported that alternate-day fasting for 4 weeks following an 8-week high-fat diet decreased the glial fibrillary acidic protein expression in the hippocampus, thereby alleviating astrocytic activation [92]. Meanwhile, some studies examining the effects of probiotics on autistic symptoms have reported that the treatment is associated with an anti-neuroinflammatory response. It has been shown that *Lactobacillus* species, administered separately or as a multistrain formulation at a dose of 1 × 10^9^ cfu/mL/drinking water for 42 days, suppressed VPA-induced IL-6 and TNF-α increases and IL-10 decreases in the brain [93]. Furthermore, in our previous study, we demonstrated that a 42-day intervention with VSL#3 at a dose of 22.5 × 10^9^ cfu/day normalized the IL-6 increase and IL-10 decrease caused by prenatal VPA exposure [38]. Additionally, autism-like behaviors and increases in IL-6 and IL-17a were observed in the offspring of mothers immunologically activated with poly(I:C); however, a probiotic mixture containing *Lactobacillus* and *Bifidobacterium* (9.89 × 10^9^ cfu/100 mL) administered during gestation prevented these symptoms [94]. Beyond these findings, our results indicating that the next-generation probiotic *Akkermansia muciniphila* was associated with reduced neuroinflammatory markers provide a unique contribution to the literature. Moreover, the fact that the neuroinflammatory marker profile in the group receiving combined treatment with *Akkermansia muciniphila* and intermittent fasting was closer to that of the healthy control group is noteworthy and supports the possibility of greater combined effects of these interventions.

Apoptosis is a fundamental neurodevelopmental process. Although no consensus exists regarding its central role in autism spectrum disorder, several experimental and post-mortem studies have suggested that alterations in apoptotic and cellular stress pathways may contribute to autism-related neurobiological changes [15]. In studies conducted on autistic brain tissue, the activation of signals associated with endoplasmic reticulum stress and an increase in pro-apoptotic markers have been detected in both the prefrontal cortex and hippocampus, suggesting that this condition may contribute to the autistic phenotype [16]. In addition to apoptosis, autophagic flux impairment is also thought to be involved in the neurobiological mechanisms associated with autism. Autophagy dysfunction not only disrupts synaptic development, dendritic spine morphology, and neuronal circuit formation but also shows a strong correlation with the intellectual disability reported in individuals with autism [95,96]. Studies have shown that, in the context of autism, both apoptotic pathway components and autophagic flux markers are significantly altered not only in brain tissue but also in peripheral cells [96,97]. Within the scope of the current study, although BAX—one of the apoptosis-related markers—showed an increasing trend with prenatal VPA exposure, the differences between groups were not statistically significant. However, the decrease in BCL-2 levels and the increase in the BAX/BCL-2 ratio suggest a shift in the balance of apoptosis-related signaling in both the prefrontal cortex and hippocampus. Importantly, alterations in BAX, BCL-2, or their ratio should not be interpreted as direct evidence of apoptosis per se; instead, these markers are commonly regarded as indicators of cellular stress and the susceptibility of cells to apoptotic signaling. Therefore, our findings are more appropriately interpreted as reflecting VPA-induced disturbances in cellular stress and survival pathways, rather than as direct evidence of apoptosis driving autistic phenotypes. Furthermore, prenatal VPA exposure reduced the levels of LC3-II, a critical marker of autophagosome formation, and Beclin-1, the most important protein associated with the initiation of autophagy, in both brain tissues. These findings—showing that apoptotic and autophagic processes are disrupted as a result of establishing a VPA-induced autism model—are consistent with the literature. Numerous studies have reported that prenatal VPA exposure is associated with alterations in apoptosis-related signaling, including reduced anti-apoptotic BCL-2 levels and increased pro-apoptotic BAX and activated caspase-3 expression across brain regions such as the prefrontal cortex, hippocampus, and cerebellum [98,99,100,101,102]. It has been shown that VPA also extensively suppresses autophagic processes by activating the mTOR signaling pathway in the prefrontal cortex and hippocampus. In VPA-treated animals, phospho-mTOR and phospho-S6 levels increased, while autophagy markers such as Beclin-1, Atg5, Atg10, and LC3-II and the percentage of cells with autophagic vacuoles decreased [102,103]. Despite the disruptive effects of VPA, evidence suggests that intermittent fasting may influence apoptosis-related signaling and autophagic flux. Intermittent fasting approaches, such as time-restricted feeding and dietary energy restriction, have been associated with increased BCL-2 levels and reduced pro-apoptotic markers, including caspase-3, BAX, and the BAX/BCL-2 ratio in peripheral and neural tissues, indicating a shift toward cellular survival and stress adaptation pathways [104,105,106]. Additionally, studies conducted in different experimental models have shown that intermittent fasting improves autophagic activity by increasing LC3-II and Beclin-1 expression, decreasing p62 accumulation, and alleviating p-AMPK/p-mTOR imbalances in brain, spinal cord, liver, and kidney tissues [105,107,108,109,110]. Furthermore, several studies have suggested that probiotic supplementation may influence apoptosis-related signaling and autophagic pathways. Species such as *Lactobacillus*, *Bifidobacterium*, and *Lactococcus*, administered individually or as multispecies formulations in experimental autism models, have been reported to reduce caspase-3 activity and histological indicators associated with apoptotic cell death [93,111]. Additionally, it has been reported that these species support autophagic flux and cellular homeostasis by modulating the expression of ATG genes associated with autophagy and inhibiting the mTORC1 signaling pathway [112,113]. Although no direct anti-apoptotic effects of *Akkermansia muciniphila* have been reported, there is significant evidence that it supports autophagic pathways, particularly in peripheral tissues. In an experimental study, *Akkermansia muciniphila* was reported to reverse COPD-induced changes in p62, p-mTOR, and LC3 expression in lung tissue [114]. In another study, it was found that Amuc_1100, an outer membrane protein of *Akkermansia muciniphila*, increased the activity of CD8^+^ T cells via autophagic pathways and enhanced antitumor immunity in lung adenocarcinoma cultures [115]. In addition, a decrease in the levels of *Akkermansia muciniphila* and its most important metabolite, propionate, has been observed in the gut microbiota composition of individuals with Alzheimer’s disease. The same study reported that propionate intervention enhances PINK1/PARKIN-mediated mitophagy in the hippocampus. Therefore, it has been suggested that *Akkermansia muciniphila* may support neuronal autophagy via propionate [44]. Considering all these data together, intermittent fasting and specific probiotic interventions may contribute to the restoration of cellular homeostasis in the context of VPA-induced disruptions in autophagic flux and apoptosis-related signaling. However, the simultaneous effects of intermittent fasting and *Akkermansia muciniphila* on neuronal autophagy and apoptosis-related pathways have not yet been directly investigated. The present study is the first to explore the anti-apoptotic and autophagy-modulating roles of intermittent fasting and *Akkermansia muciniphila* in a VPA-induced autism model, offering a novel perspective on their potential combined effects in the re-regulation of autophagic flux and cellular survival pathways.

GAD67—one of the two isoforms of the rate-limiting glutamate decarboxylase enzyme that catalyzes the conversion of L-glutamate to GABA—is critical for maintaining physiological GABA levels [116], and adequate expression of this enzyme is necessary for maintaining the excitatory-inhibitory (E/I) balance. In particular, GAD67 deficiency in GABAergic interneurons, especially parvalbumin-positive neurons, leads to excessive activation of glutamatergic transmission, causing disruption of the E/I balance and increased excitability [117]. The chromosome encoding the GAD67 isoform has been reported to be a susceptibility locus associated with autism [116]. Consistent with our previous study, we found that prenatal VPA exposure decreased GAD67 levels in the prefrontal cortex and hippocampus [68], while the considered treatments generally improved GAD67 levels. This is an important finding, indicating that both intermittent fasting and *Akkermansia muciniphila* can calm excitation. Ketone levels—particularly β-hydroxybutyrate—increase during intermittent fasting, and increased ketones may maintain the E/I balance by upregulating GABA [118,119]. A study reported that 6 weeks of alternate-day fasting restored inhibitory synaptic transmission by improving presynaptic GABA release in aged mice [120]. Although there is evidence that intermittent fasting may support GABA production, no study in the current literature has reported a direct effect of fasting on GAD levels. However, it has been noted that probiotics can produce GABA from glutamate via GAD, and that GABA generated by bacteria may modulate brain functions via the vagus nerve or circulation [121,122]. This hypothesis is also strongly supported by experimental autism studies showing that species such as *Bifidobacterium* and *Lactobacillus* can increase GABA levels, GABA receptor expression, and GABA/glutamate ratios [123,124]. In fact, a strong view is increasingly emerging that the main inhibitory neurotransmitter in the central nervous system may be GABA, which originates from the gut microbiota [125]. It has been demonstrated that *Akkermansia muciniphila* can produce physiologically significant amounts of GABA in the acidic intestinal environment [126], and the relative abundance of *Akkermansia muciniphila* in the gut microbiota has been reported to be positively associated with GABA levels in the serum, cortex, and hippocampus [127,128]. Additionally, a three-strain probiotic containing *Akkermansia muciniphila* provided beneficial effects by increasing the GABA/glutamate ratio when administered to epileptic mice for 28 days [129]. GABA production is a mechanism that supports the adaptation of bacteria to acidic stress, and it plays a critical role in sustaining colonization in the gastrointestinal system and survival under acidic fermentation conditions [121]. Therefore, some bacteria contain genes encoding GAD, which is responsible for GABA production [121]. According to our results, it is plausible that *Akkermansia muciniphila* also contributes to GABA-related signaling. Even though a few recent studies have suggested that intermittent fasting and *Akkermansia muciniphila* interventions may affect GABA levels, we are the first to demonstrate that they increase GAD67 levels. Therefore, the increase in GAD67 levels may represent one potential pathway through which these interventions exert neurobiological effects.

Parvalbumin-positive interneurons are characterized by their fast-spiking properties and targeted projections to their soma or axon initial segment [130]. These inhibitor cells play a fundamental role in the emergence of high-frequency gamma oscillations, which are associated with cognitive functions such as attention, learning, memory, social behavior, and perception [24,131]. Our findings demonstrated that prenatal VPA exposure significantly decreased parvalbumin levels in both brain regions. This is consistent with studies reporting decreased density of parvalbumin-positive neurons or parvalbumin expression in brain regions such as the prefrontal cortex, hippocampus, striatum, and amygdala in the prenatal VPA-induced autism model [68,132,133,134]. Parvalbumin-positive neurons require more energy than other neuronal subtypes to maintain their rapid firing capacity, making them sensitive to stressful environmental conditions [135]. Therefore, it is highly likely that the inflammatory processes triggered by VPA weaken parvalbumin immunoreactivity. Decreased or dysfunctional parvalbumin causes impairments in calcium buffering, action potential timing, excitatory neuronal activity, and gamma oscillations in the medial prefrontal cortex, negatively affecting social cognitive processes [136,137]. In contrast, certain treatments have been successful at increasing parvalbumin levels, particularly in the prefrontal cortex. A limited number of studies in the literature have highlighted the relationship between intermittent fasting or probiotic treatments and the parvalbumin density in neuronal cells. The effect of intermittent fasting on parvalbumin levels has only been reported in a recently published study involving an Alzheimer’s model, where it was reported that alternate-day fasting in 5xFAD mice for 4 months mitigated the decrease in the number of parvalbumin-positive neurons in the retrosplenial granular, parietal, and somatosensory cortices [138]. While it has been shown that administration of a multistrain probiotic containing *Bifidobacterium* and *Lactobacillus* species during pregnancy alleviated parvalbumin deficiency in the prefrontal cortex of adult offspring [94], a similar effect of *Akkermansia muciniphila* on parvalbumin is reported for the first time in the present study. Taken together, these findings suggest that both intermittent fasting and *Akkermansia muciniphila* may modulate parvalbumin-mediated inhibitory circuits disrupted by prenatal VPA exposure, and that parvalbumin interneurons may be a potential therapeutic target in neurodevelopmental disorders associated with autism.

It is thought that another critical condition in the etiology of autism is the disruption of intestinal barrier function. The intestinal epithelial barrier controls the absorption of intestinal contents into the bloodstream and prevents harmful compounds from leaking into the systemic circulation. Thus, it suppresses the inflammatory response and maintains gastrointestinal and systemic homeostasis. However, if this barrier is compromised (leaky gut), these functions are disrupted and microbial products, toxins, and other antigens can cross into the bloodstream. It is widely believed that this mechanism contributes to neuroinflammation and neurological symptoms via the gut–brain axis [139]. Zonulin is the only well-defined molecule that temporarily disrupts tight junction proteins between epithelial cells. It is a multidomain protein and is released into the intestinal lumen by enterocytes upon stimulation by intestinal pathogens and gluten [140]. Accumulating evidence suggests that serum zonulin levels, an important indicator of intestinal permeability, are elevated in autism [141,142,143,144]. Zonulin has also been found to be positively associated with the severity of behavioral symptoms in children with autism [143]. Therefore, some studies argue that zonulin could potentially be considered a biomarker for assessing the severity of autism [144]. There are very few studies focusing on the effects of prenatal VPA exposure on gut barrier function. One of these few studies reported that VPA administration significantly triggered intestinal permeability in male offspring [145]. In contrast, *Akkermansia muciniphila* has been shown to attach to epithelial cells using the intestinal mucus layer and to strengthen intestinal barrier function by increasing the expression of tight junction proteins [146]. A recently published study reported that *Akkermansia muciniphila*, administered at a dose of 5 × 10^7^ cfu/day for three days, enhanced the intestinal barrier by decreasing zonulin levels [147]. This is the only literature report indicating that *Akkermansia muciniphila* improves zonulin levels, and is consistent with our findings. On the other hand, no literature finding examining the effect of intermittent fasting on intestinal permeability has been reported. Only one study reported that serum zonulin levels decreased significantly during fasting compared to postprandial period [148]. Differently from this, we demonstrated that subchronic intermittent fasting improved serum zonulin levels. Inflammation induced by changes in the gut microbiota may compromise intestinal barrier function. Proinflammatory cytokines destroy the mucins that form the mucus layer [149]. Therefore, the reduction in zonulin levels observed with intermittent fasting and *Akkermansia muciniphila*, both of which exert anti-inflammatory effects, may suggest a potential alleviating effect on intestinal permeability. However, stating that these interventions definitively restore gut barrier integrity would be overly assertive. There are ongoing discussions in the literature regarding the specificity of zonulin-based ELISA measurements [150]. Moreover, zonulin alone may not fully reflect functional intestinal permeability. Future studies combining functional permeability assays such as the FITC-dextran translocation test, lactulose/mannitol ratio, and Ussing chamber experiments with tissue-level analyses of tight junction proteins (e.g., occludin, claudin-1) would provide more direct evidence supporting conclusions about barrier function.

This study has several limitations that should be considered when interpreting the findings. First, although the sample size was consistent with previous prenatal VPA studies and supported by a priori sensitivity analysis, the relatively small group size may limit the generalizability of the results. Second, metabolic and systemic parameters (e.g., glucose, insulin, lipid profile) were not evaluated, which limits conclusions regarding the broader metabolic mechanisms underlying the observed neurobiological improvements. In addition, autophagy was assessed using LC3-II and Beclin-1 protein levels, and direct assessment of autophagic flux was not performed, which should be considered when interpreting the autophagy-related findings. Third and most importantly, there is no direct assessment of gut microbiota composition, *Akkermansia muciniphila* colonization, and microbial metabolite production (e.g., short-chain fatty acids). Fecal sampling was not performed, and strain-specific colonization was not verified. Furthermore, mechanistic gut-barrier markers such as tight junction proteins (occluding and claudin-1), mucus-layer markers (MUC2), and circulating endotoxin-related markers (LPS or LBP) were not evaluated. Therefore, although the findings are consistent with gut–brain-related mechanisms, causal attribution to microbiome remodeling is not possible. Future studies integrating microbiome profiling and metabolomic analyses are required to clarify these mechanisms. Finally, only male offspring were included due to the stronger and more reproducible phenotype in the VPA model; thus, the findings cannot be generalized to females, and future studies should also incorporate sex-specific analyses.

## 5. Conclusions

This study provides a comprehensive framework by examining the multidimensional effects of intermittent fasting and *Akkermansia muciniphila* on behavioral outcomes, neuroinflammation, autophagic and GABAergic markers, and intestinal permeability within the same experimental setting in a prenatal VPA-induced autism model. The observation that both interventions, applied individually and in combination, improved biomarkers relevant to autism-associated neurobiology, including GAD67, parvalbumin, and zonulin, represents a key strength of the study. On the other hand, the study relied on a single experimental model, and the underlying molecular mechanisms were not investigated at a causal level. In addition, in-depth characterization of gut microbiota composition was beyond the scope of the present work. Future studies employing multiple autism models and integrating microbiome-focused, metabolic, and sex-specific analyses will be essential to further clarify the underlying mechanisms and strengthen the translational relevance of these findings.

Further research should adopt more comprehensive mechanistic approaches to better define the biological pathways underlying the observed effects. In particular, integrative omics strategies—including metabolomics, transcriptomics, and proteomics—may provide deeper insight into microbiota-host-brain interactions and metabolite changes associated with intermittent fasting and *Akkermansia muciniphila*. Multi-omics approaches have previously been shown to provide valuable information on metabolic and molecular alterations in neurodevelopmental disorders [151], and the application of similar strategies in VPA-induced autism models may help identify the molecular mediators and metabolic signatures linking gut microbiota modulation to brain function. Nevertheless, our findings suggest that metabolic and microbial-based interventions offer promising complementary strategies that could target the multidimensional biology of autism.

## Data Availability

The raw data supporting the conclusions of this article will be made available by the authors on request.

## Abbreviations

The following abbreviations are used in this manuscript:

DSM	Diagnostic and statistical manual of mental disorders
ARRIVE	Animal research: reporting of in vivo experiments
IL	Interleukin
TNF	Tumor necrosis factor
IFN	Interferon
BCL	B-cell lymphoma 2
BAX	Bcl-2-associated X protein
LC	Light chain
ATP	Adenosine triphosphate
AMPK	Adenosine monophosphate-activated protein kinase
mTOR	Mammalian target of rapamycin
ULK	Unc-51-like autophagy activating kinase
Atg	Autophagy-related
PI3K	Phosphoinositide 3-kinase
GABA	γ-aminobutyric acid
TLR	Toll-like receptor
PTEN	Phosphatase and tensin homolog
PINK	PTEN-induced putative kinase
COPD	Chronic obstructive pulmonary disease
Poly(I:C)	Polyinosinic: polycytidylic acid
